# The type VI secretion system protein AsaA in *Acinetobacter baumannii* is a periplasmic protein physically interacting with TssM and required for T6SS assembly

**DOI:** 10.1038/s41598-019-45875-9

**Published:** 2019-07-01

**Authors:** Lei Li, Yi-Nuo Wang, Hong-Bing Jia, Ping Wang, Jun-Fang Dong, Juan Deng, Feng-Min Lu, Qing-Hua Zou

**Affiliations:** 1 Department of Microbiology, School of Basic Medical Sciences, Peking University Health Science Center, Beijing, 100191 China; 2 National Center for Health Professions Education Development, Beijing, 100191 China; 3 Department of clinical laboratory, China-Japan friendship hospital, Beijing, China

**Keywords:** Bacterial secretion, Bacterial pathogenesis, Bacterial secretion, Bacterial pathogenesis

## Abstract

Type VI secretion system (T6SS) is described as a macromolecular secretion machine that is utilized for bacterial competition. The gene clusters encoding T6SS are composed of core *tss* genes and *tag* genes. However, the clusters differ greatly in different pathogens due to the great changes accumulated during the long-term evolution. In this work, we identified a novel hypothetical periplasmic protein designated as AsaA which is encoded by the first gene of the T6SS cluster in the genus *Acinetobacter*. By constructing *asaA* mutant, we delineated its relative contributions to bacterial competition and secretion of T6SS effector Hcp. Subsequently, we studied the localization of AsaA and potential proteins that may have interactions with AsaA. Our results showed that AsaA in *Acinetobacter baumannii* (*A*. *baumannii*) localized in the bacterial periplasmic space. Results based on bacterial two-hybrid system and protein pull-down assays indicated that it was most likely to affect the assembly or stability of T6SS by interacting with the T6SS core protein TssM. Collectively, our findings of AsaA is most likely a key step in understanding of the T6SS functions in *A*. *baumannii*.

## Introduction

*Acinetobacter baumannii* (*A*. *baumannii*) is an important Gram-negative opportunistic pathogen which is commonly found in soil, water and on human skin. As an important species of *Acinetobacter*, *A*. *baumannii* is one of the most commonly isolated Gram-negative bacteria in clinical isolates. *A*. *baumannii* infections are becoming more and more difficult to treat due to multi-and pan-drug resistant strains^1^.

The type VI secretion system (T6SS) is a recently describe specialized secretion machinery used by a wide variety of Gram-negative bacteria to target against both prokaryotic and eukaryotic competitors^2–5^. The system consists of several proteins forming a needle like structure^6^ that grants Gram-negative bacteria the capacity to translocate substrates such as phospholipases, peptidoglycan hydrolases, nucleases, and membrane pore-forming proteins to neighboring cells in order to kill^7^. It has been well demonstrated that T6SS can mediate interbacterial competions and thus give the bacteria growth advantages to settle in natural habitats^8^. It is worth mentioning that more recently, some studies revealed that the competitions between bacteria mediated by T6SS can also foster horizontal gene transfer (HGT)^9,10^. A study from Cooper *et al*. indicated that contact-dependent neighbor killing by T6SS may be a widespread contributor to HGT, and for *Acinetobacter* in particular, killing-enhanced HGT may play a key role in the emergence of clinically pervasive MDR ‘super-bug’ strains^11^. Since there is a high incidence of antibiotic resistance in *A*. *baumannii*, a comprehensive study on the T6SS of *A*. *baumannii* may give us important clues on the pathogenisis and acquisition of antibiotic resistance of this bacteria.

The clusters encoding T6SS is composed of 13 core *tss* genes (type six subunit genes, *tssA*~*tssM*) and a variable number of *tag* genes (T6SS-associated genes) at least^6,12^. The components of T6SS are assembled in an orderly manner into a macromolecular machine, which is composed of two components: the tail-like structure and the membrane-spanning structure^13^. The extracellular components of the T6SS, Hcp and VgrG, form a needle-like injection device closely resembling the bacteriophage tail. At the same time, Hcp is one of the main components secreted by T6SS apparatus, and the presence of secreted Hcp in culture supernatants is a well-established molecular marker of a functional T6SS^14^. The T6SS-encoding cluster of *A*. *baumannii* harbors more than 20 different genes. The first gene termed *asaA* (*Acinetobacter* type six secretion system-associated *A* gene) is specific to the genus *Acinetobacter* and is highly conserved in *A*. *baumannii*. However, till now it has not been experimentally tested for its contributions to the pathogenesis in *A*. *baumannii*. In the studies performed by Weber *et al*. and Ringel *et al*., an AsaA homolog (ACIAD2693) in *A*. *baylyi* ADP1 was found, and results showed that it is important for T6SS function^10,12^. However, great difference exist in the amino acid sequences between the AsaA in *A*. *baumannii* and *A*. *baylyi*. In this study, to have a better understanding the function of AsaA in *A*. *baumannii*, we studied the effect of AsaA on T6SS secretion and potential proteins that may have interactions with AsaA in *A*. *baumannii*.

## Results

### AsaA is *Acinetobacter* specific and highly conserved in the species *A*. *baumannii*

Previous studies have described the gene cluster of T6SS in *A*. *baumannii*^12,15^. The cluster is about 20 kb, consisting of more than 20 T6SS genes. *asaA* is the first gene of the T6SS cluster. In this work, we used strain ATCC 17978 as a representative of *A*. *baumannii*. In a BLAST comparison of the amino acid sequences of AsaA homologues, AsaA only present in the genus *Acinetobacter*, and the homology of the AsaA amino acid sequence differs between different species. The AsaA of *A*. *baumannii* shares 87.8–92.6% similarity with that in the species *A*. *pittii*, *A*. *calcoaceticus* and *A*. *nosocomialis*, with a much more lower similarity 46.8% and 57.8% with *A*. *baylyi* and *A*. *indicus*. In the species *A*. *baumannii*, it is highly conserved among different strains with different sequence types. The amino acid sequence of AsaA in ATCC 17978 shares 99.1–100% similarity with the other *A*. *baumannii* strains (Table S3).

### AsaA is required for bacterial competition

*asaA* was knocked out from the *A*. *baumannii* ATCC 17978 chromosome and the mutant was named *ΔasaA*. Complementation using pTrc99A which is a useful vector for the expression of unfused and fused proteins in *E*. *coli* was performed to generate *CΔasaA*^16^. To investigate whether *asaA* knock-out would affect the growth of *A*. *baumannii*, the *ΔasaA*, *CΔasaA* and wild type (WT) 17978 were tested for the growth rates in LB. As shown in Supplementary Fig. 1, there were no significant difference between different strains. So knocking out *asaA* did not affect the growth of *A*. *baumannii* in LB.

**Figure 1:**
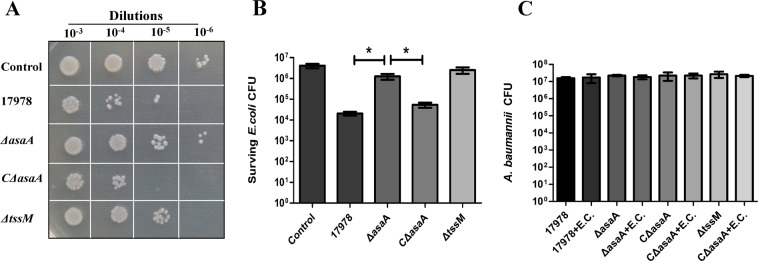
Competition between *A*. *baumannii* strains and *E*. *coli*. (**A**) Semi-quantitative assessment of surviving *E*. *coli* in growth medium (control) or with WT 17978, *ΔasaA*, *CΔasaA* and *ΔtssM* strains. (**B**) Quantitative assessment of the survival of *E*. *coli* and *A*. *baumannii*. Values are the means ± standard deviation from three repeats. Error bars represent standard error of the mean of the biological replicates. *Indicates a significant difference between *E*. *coli* CFU observed with different *A*. *baumannii* strains. (**C**) The survival of *A*. *baumannii* among different groups.

A recent study found that *A*. *baumannii* ATCC 17978 can utilize its T6SS to compete with *E*. *coli*^15^. To determine whether AsaA is required for the competition, we used *E*. *coli* strain JM109/pK18*mob*, which contained a kanamycin resistant plasmid pK18*mob*, as a target for bacterial competition assays. TssM is a core component of T6SS. Previous study has shown deletion of *tssM* in *A*. *baumannii* can completely abolish its ability to outcompete *E*. *coli*^15^. So we used *ΔtssM* mutant as a negative control. WT 17978, *ΔasaA*, *CΔasaA* or *ΔtssM* were incubated with *E*. *coli* and surviving *E*. *coli* were calculated. The results revealed that there were significant differences among the competition ability of these bacteria. Compared with WT 17978 and *CΔasaA*, the number of surviving *E*. *coli* is much higher in both *ΔasaA* and *ΔtssM* groups than in WT 17978 and *CΔasaA* groups, suggesting that deletion of *asaA* resulted in a decreased competitive ability in WT 17978 (Fig. 1A). Quantitatively, bacterial mixtures were plated onto kanamycin resistant and non-resistant plates to culture *E*. *coli* and total bacteria. As shown in Fig. 1B, similar to *ΔtssM*, *ΔasaA* mutant has a low ability to kill *E*. *coli* and there were no significant differences in the survival of *E*. *coli* strains between *ΔtssM* and *ΔasaA* mutants. In contrast, the survival of *E*. *coli* strains in the WT 17978 and *CΔasaA* groups decreased significantly (Fig. 1B). At the same time, the survival of *A*. *baumannii* were not influenced by *E*. *coli* and there were no significant differences in the survival of *A*. *baumannii* among different groups (Fig. 1C). These results suggest that AsaA is critical for bacterial competition in *A*. *baumannii* ATCC 17978.

### AsaA is required for Hcp secretion

**Figure 2:**
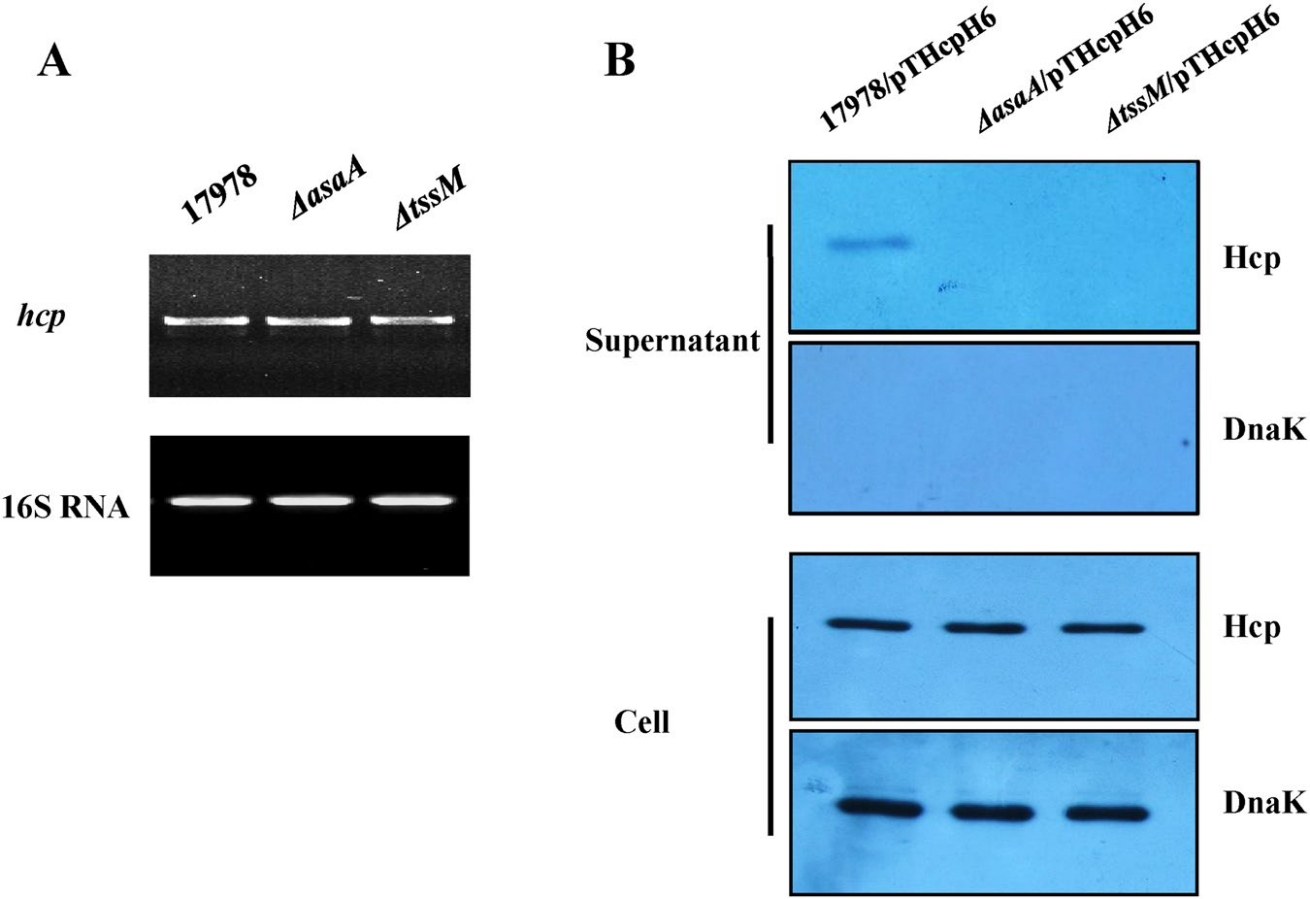
AsaA is critical for the secretion of Hcp in *A*. *baumannii* ATCC 17978. (**A**) *hcp* mRNA levels tested by semi-quantitative RT-PCR. The 16S rRNA gene was used as an internal control. (**B**) The presence of Hcp in total cell lysates and concentrated culture supernatants (secreted protein) in cultures of 17978/pTHcpH6, *ΔasaA*/pTHcpH6 and *ΔtssM*/pTHcpH6 derivatives by Western blot. The presence of Hcp-His_6_ and DnaK (internal control) was detected by anti-His_6_ and anti-DnaK monoclonal antibody.

As Hcp secretion reflects the functionality of the T6SS, detection of secreted Hcp in the culture supernatants is a marker of a functional T6SS. In order to explore the mechanisms of AsaA involved in bacterial competition, we examined whether AsaA is involved in the secretion of Hcp. RT-PCR analysis showed that *hcp* were being actively transcribed in the *ΔasaA* mutant cultured in LB medium (Fig. 2A). To further investigate whether AsaA is involved in Hcp secretion, we performed Western blot assays to test the secretion of Hcp in the *ΔasaA* background. For this purpose, the recombinant plasmid pTHcpH6 containing the *hcp* gene and the 6× His-tag coding sequence, was introduced into different strains, resulting in the strains named 17978/pTHcpH6, *ΔasaA*/pTHcpH6 and *ΔtssM*/pTHcpH6, respectively (Table S1). Hcp were present in the whole cell lysates in all the three strains (Fig. 2B), at the same time, it is present in the supernatant of 17978/pTHcpH6 but absent in *ΔasaA*/pTHcpH6 or *ΔtssM*/pTHcpH6, suggesting that deletion of *asaA* affected the secretion of Hcp (Fig. 2B). These results indicated that AsaA is critical for Hcp secretion.

### AsaA located in the periplasm of *A*. *baumannii* cells

**Figure 3:**
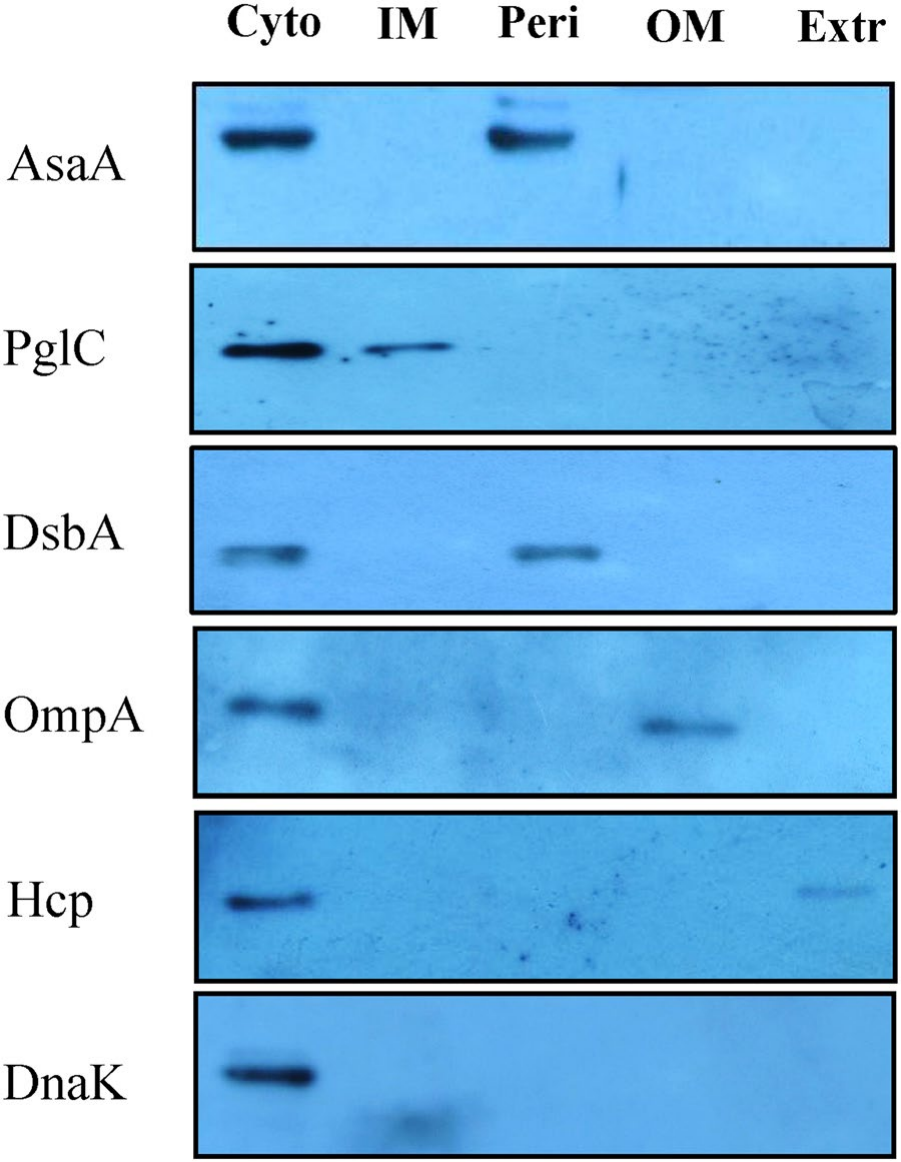
Subcellular localization of AsaA as determined. Cellular fractionation studies of AsaA in cytoplasmic (Cyto), periplasmic (Peri), inner membrane (IM), outer membrane (OM) and extracellular (Extr) fractions of *A*. *baumannii* with PglC, DsbA, OmpA, Hcp and DnaK as controls.

The protein encoded by *asaA* in *A*. *baumannii* ATCC 17978 was annotated as conserved hypothetical protein^17^. It has 230 amino acids (aa). We performed bioinformatics analysis on AsaA and failed to find additional information about its biochemical function and any known domain or motif. According to the amino acid sequence analysis of the SignalP program (http://www.cbs.dtu.dk/services/SignalP/) and the LipoP program (http://www.cbs.dtu.dk/services/LipoP/), AsaA has a signal fragment and it may be a secreted protein, but not a lipoprotein. This suggested that AsaA might be located in the periplasm or secreted outside the cells. To determine the cellular location of AsaA, we constructed a *ΔasaA*/pTasaAH6 strain, which expressed AsaA with a 6× His-tag. The total cell lysates, inter membrane, periplasmic, outer membrane and extracellular protein fractions of *ΔasaA*/pTasaAH6 cultured at logarithmic growth phase were prepared. As shown in Fig. 3, using the inner membrane protein PglC, the periplasm protein DsbA, the outer membrane protein OmpA, the extracellular protein Hcp, and the cytoplasm protein Dnak as controls, we found that AsaA is present in the periplasmic and total cell lysates but absent in the extracellular, inter membrane or outer membrane protein fractions, indicating that AsaA located in the periplasm of *A*. *baumannii*.

### AsaA interacts with TssM

**Figure 4:**
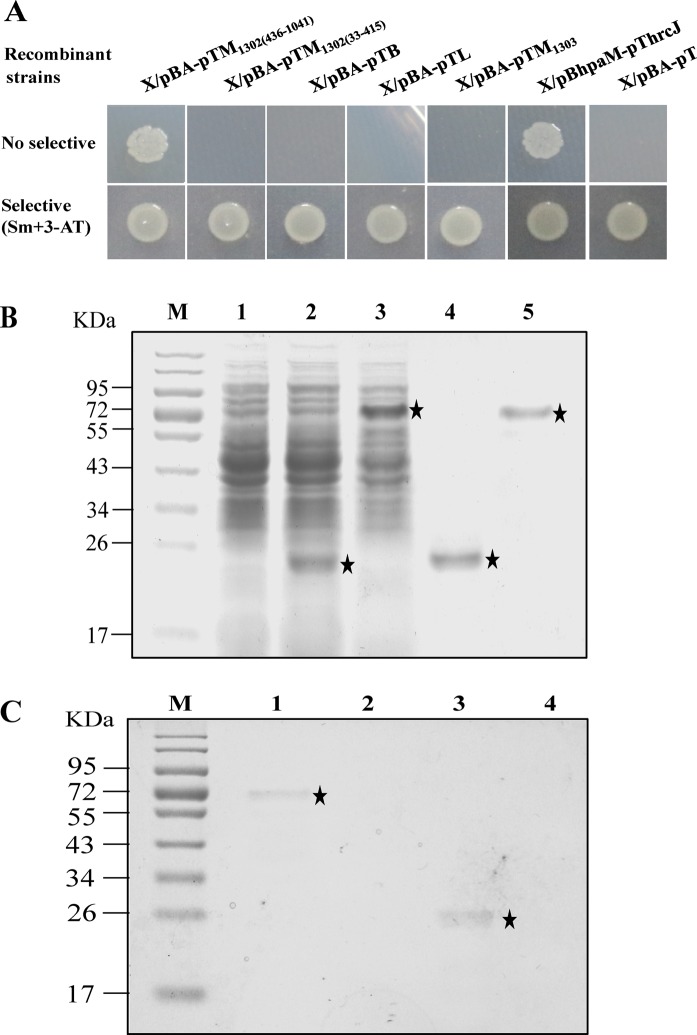
AsaA interacts with the periplasmic domains of TssM. (**A**) Bacterial two-hybrid assays. TssM_1302(436–1041)_, TssM_1302(33–415)_, TssB, TssL and TssM_1303_ fused to vector pTRG were expressed in combination with AsaA fused to vector pBT in the reporter strain, respectively. These strains were grown on non-selective and dual-selective medium. The X/pBhpaM-pThrcJ strain was used as positive controls, the X/pBA-pT strain was used as negative control. (**B**) His_6_-tagged fusion proteins were over expressed and purified. Lanes: 1, crude BL21/pET30a extract; 2, crude BL21/pET30a-AsaA extract induced with IPTG; 3, crude BL21/pET30a-TssM_1302(436–1041)_ extract induced with IPTG; 4, affinity-purified His_6_-AsaA protein; 5, affinity-purified His_6_-TssM_1302(436–1041)_ protein; M, molecular mass marker. (**C**) Pull-down assays. Lanes: 1, pull-down of His_6_-TssM by immobilized His_6_-AsaA; 2, His_6_-TssM mixed with streptavidin sepharose beads (negative control); 3, pull-down of His_6_-AsaA by immobilized His_6_-TssM; 4, His_6_-AsaA mixed with streptavidin sepharose beads (negative control); M, molecular mass marker.

Based on the facts that AsaA located in the bacterial periplasm and takes part in Hcp secretion but not involved in Hcp expression, we presumed that AsaA might physically interacts with the T6SS components. To this end, we performed bacterial two-hybrid system to investigate the possibility of the physically interactions between AsaA and a series of the T6SS core proteins: TssB, TssL and TssM. In ATCC 17978, the gene encoding TssM was divided into two open reading frames, *A1S_1302* and *A1S_1303*^18^. *A1S_1302* encodes the N-terminus and the middle part of TssM (we named this part TssM_1302_). *A1S_1303* encodes the C-terminus of TssM (we named this part TssM_1303_). In this study, we tested the interactions between AsaA with TssM_1302(33–415)_ (the 33 to 415 amino acids of TssM_1302_ which composite the inter membrane domain of TssM), TssM_1302(436–1041)_ (the 436 to 1041 amino acids of TssM_1302_ which composite the periplasmic domain of TssM) and TssM_1303_. The open reading frame of *asaA* excluding the N-terminal leader was fused to pBT, yielding a plasmid named as pBA. The open reading frames encoding TssB, TssL, TssM_1303_, TssM_1302(33–415)_ and TssM_1302(436–1041)_ were fused to pTRG, yielding recombinant plasmids named as pTB, pTL, pTM_1303_, pTM_1302(33–415)_ and pTM_1302(436–1041)_, respectively. Finally, the plasmids were co-introduced into XL1-Blue MRFʹ strain. For confirmation, all the resulting recombinant strains were tested on dual-selective medium containing 3-AT and streptomycin. If interactions between the proteins occur, the recombinant strains can obtain the ability to grow on dual-selective medium due to the activation of *HIS3*-*addA* reporter genes. Our results showed that only the X/pBA-pTM_1302(436–1041)_ can grow well on dual-selective medium, in contrast, the other recombinant strains can’t grow on dual-selective medium (Fig. 4A), implying that physical interactions existed between AsaA and the periplasmic domain of TssM in the reporter strain.

To validate the interaction between AsaA and TssM_1302(436–1041)_, the pull-down biotinylated protein-protein assay was further performed. The AsaA_25–230_ (from the 25^th^ to 230^th^ aa) and TssM_1302(436–1041)_ encoding sequences were cloned into the expression vector pET-30a to produce the recombinant His-tagged proteins AsaA-His_6_ and TssM-His_6_ (Fig. 4B, full-length gel was presented in Supplementary Fig. 2). AsaA-His_6_ was immobilized on sepharose beads, and then pull-down assays for TssM-His_6_ was performed. As shown in Fig. 4C (full-length gel was presented in Supplementary Fig. 3), the AsaA-His_6_ protein was able to capture TssM-His_6_ protein (lane 1). Vice versa, TssM-His_6_ protein immobilized on streptavidin sepharose beads was also able to capture AsaA-His_6_ protein (lane 3). So, AsaA-His_6_ and TssM-His_6_ can capture each other. The results indicated that AsaA interacts directly with TssM_1302(436–1041)_.

**Figure 5:**
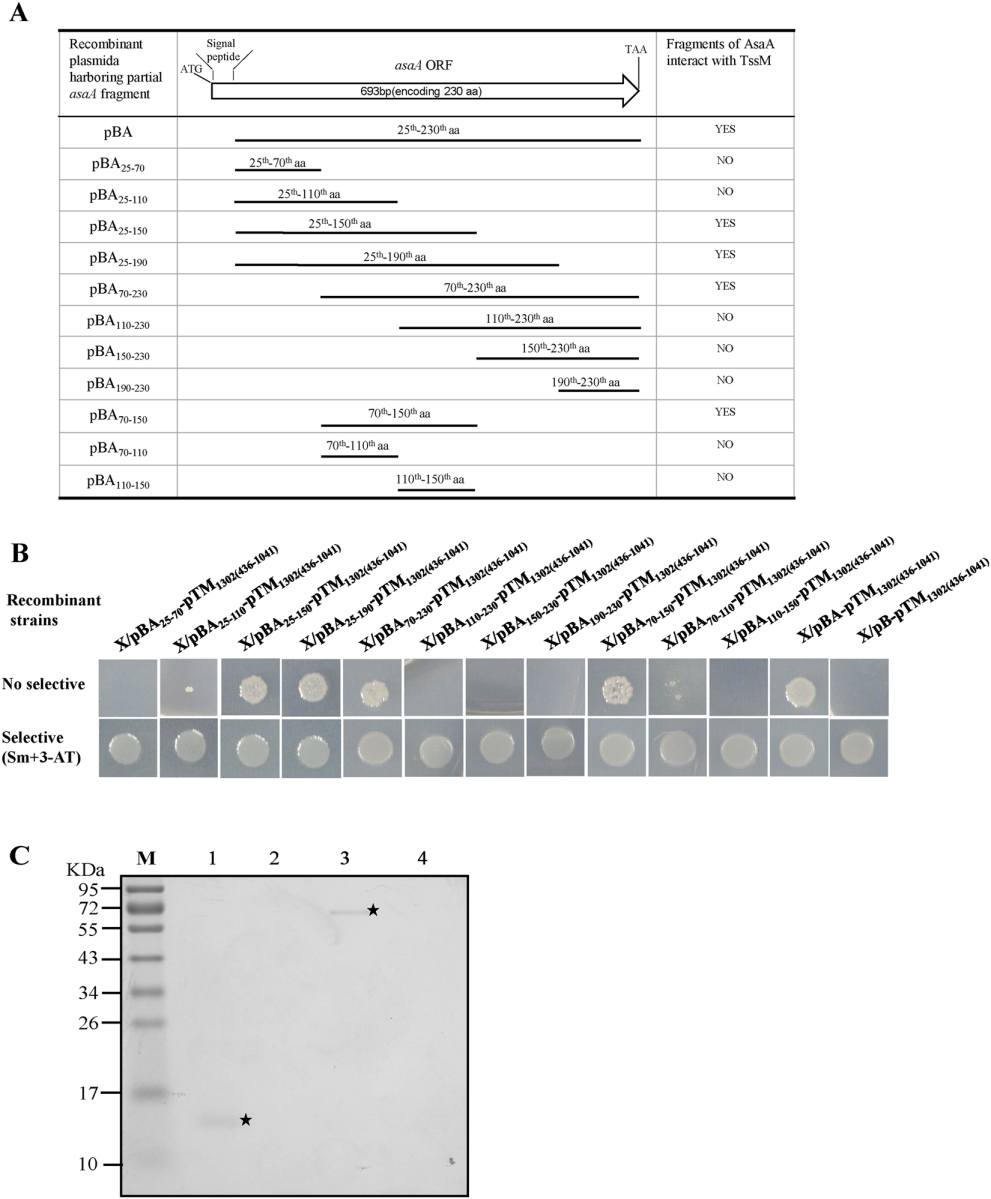
Determination of the AsaA fragment required for the interaction with TssM. (**A**) Different AsaA fragments tested for the interaction with TssM. YES, fragment can interact with TssM_1302(436–1041)_; NO, fragment can’t interact with TssM_1302(436–1041)_. (**B**) Bacterial two-hybrid assays. The X/pBA-pTM_1302(436–1041)_ strain was used as positive controls, the X/pB-pTM_1302(436–1041)_ strain was used as negative control. (**C**) Pull-down assays. Lanes: 1, pull-down of His_6_-AsaA_70–150_ by immobilized His_6_-TssM; 2, His_6_-AsaA_70–150_ mixed with streptavidin sepharose beads (negative control); 3, pull-down of His_6_-TssM by immobilized His_6_-AsaA_70–150_; 4, His_6_-TssM mixed with streptavidin sepharose beads (negative control); M, molecular mass marker.

We then tried to find the exact domains of AsaA that interacts with TssM_1302(436–1041)_. Since the first 24 amino acids of AsaA encode a signal peptide, we used the truncated AsaA excluding the N-terminal signal peptide sequence for testing. DNA fragments as shown in Fig. 5A encoding the different domains of AsaA (the 25^th^–70^th^ aa, 25^th^–110^th^ aa, 25^th^–150^th^ aa, 25^th^–190^th^ aa, 70^th^–230^th^ aa, 110^th^–230^th^ aa, 150^th^–230^th^ aa and 190^th^–230^th^ aa) were amplified and fused to pBT, respectively. Then, the recombinant plasmids and pTM_1302(436–1041)_ were co-introduced into XL1-Blue MRFʹ strain, respectively. All the resulting recombinant strains were tested on dual-selective medium. Only the strains which harbored the plasmid pBA_25–150_-pTM_1302(436–1041)_, pBA_25–190_-pTM_1302(436–1041)_ and pBA_70–230_-pTM_1302(436–1041)_ could grow on the dual-selective medium but others could not (Fig. 5B). This indicated that the 70^th^–150^th^ aa of AsaA were crucial for the interaction. We further tested the interactions of the 70^th^–150^th^ aa, 70^th^–110^th^ aa, and 110^th^–150^th^ aa of AsaA with TssM_1302(436–1041)_. Only the strain containing pBA_70–150_-pTM_1302(436–1041)_ could grow on the selective agar plates as shown in Fig. 5B. The pull-down biotinylated protein-protein assay was performed for further validation. As shown in Fig. 5C, TssM-His_6_ protein was able to capture AsaA_70–150_-His_6_ protein (lane 1). Vice versa, AsaA_70–150_-His_6_ was also able to capture TssM-His_6_ protein (lane 3). Our results indicated that the peptide of AsaA consisting of aa from the 70^th^ to the 150^th^ is sufficient to interact with the periplasmic domain of TssM.

## Discussion

Our knowledge about the molecular architecture and the function of T6SS has great strides. For *A*. *baumannii*, several studies have been performed to elucidate its functions. It has been proven that the T6SS of *A*. *baumannii* is responsible for bacterial competition and is also implicated in host colonization^12,15,19–21^. However, for the functions of the proteins in T6SS, only a few were approved experimentally, including VgrG which contributes to both virulence and antimicrobial resistance in *A*. *baumannii* ATCC 19606^21^ and apeptidoglycan hydrolase, named TagX, which is conserved in the genus *Acinetobacter* and is essential for Hcp secretion^12^. There were still a lot need to be elucidated about the T6SS in *A*. *baumannii*.

In this study, we made a detailed study on the function of AsaA. AsaA is encoded by the first gene within the T6SS cluster. By comparative bioinformatics analysis, we found AsaA is specific to the genus *Acinetobacter* and is highly conserved in the species *A*. *baumannii*. Studies on the function of the highly conserved and *Acinetobacter* specific T6SS protein AsaA can help us learn more about the pathogenesis of *A*. *baumannii*. We used ATCC 17978 as a representative of *A*. *baumannii* to study whether AsaA is critical for bacterial competition. The competition ability of ATCC 17978 varied with different prey cells. Guillermo *et al*. showed ATCC 17978 can compete against *E*. *coli* DH5α^15^, while Weber *et al*. showed that ATCC 17978 is unable to utilize its T6SS for antibacterial activity against *E*. *coli* MG1655^18^. We postulated that different *E*. *coli* strains may have different immunity proteins and thus resulted in different competition abilities. In this study, we found ATCC 17978 can well compete *E*. *coli* JM109 and AsaA is critical for the competition.

Our results demonstrated that AsaA localizes in the periplasm, where it interacts with the periplasmic domain of TssM by a 80 aa peptide from the 70^th^ to 150^th^ aa, forming a two protein complex. AsaA is not participated in the regulation of the Hcp expression but is essential for Hcp secretion in *A*. *baumannii* ATCC 17978. At this stage, AsaA played an important role in the T6SS, but its precise roles remain to be determined. However, based on the facts that (1) mutants of AsaA and TssM showed similar phenotypes in bacterial competition and Hcp secretion; (2) similar to *tssM*, *asaA* is within the T6SS gene cluster; and (3) the TssM_1302(436–1041)_ extends into the periplasm and interact with AsaA physically, we presume that AsaA is most likely to be a structural component of the T6SS. The interaction with TssM may consolidate the structure of T6SS and thus facilitate the transportation of effectors.

The T6SS structural components are encoded by T6SS cluster, which are probably acquired by horizontal gene transfer during evolution. It has been shown that *A*. *baumannii* T6SS clusters appear to be conserved amongst sequenced *A*. *baumannii* strains with the exception that a portion of the gene cluster is inverted^15^. However, the clusters have undergoing great changes during the long-term evolution. A main difference from other bacteria is that the genus *Acinetobacter* do not produce a readily identifiable homolog of core-components TssJ^18^, which has been shown to interact with the extreme C-terminus of TssM. Whether AsaA can replace TssJ is of interest. In this study, we found that: (1) AsaA does not interact with TssM_1303_, which encode the C-terminus of TssM, but interact with TssM_1302,_ which encode the N-terminus and the middle part of TssM; (2) AsaA localizes in periplasm, while TssJ localizes in outer membrane; and (3) secondary structure of AsaA is predicted to be alpha helix, while TssJ is mostly beta-folded. Base on these facts, we presume that AsaA is unlikely a replacement protein for TssJ^22^.

Contact-dependent growth inhibition (CDI) system are alternative war machines doing the same job as T6SS, i.e. fight against neighbouring cells and oucompeting them. They are found on the cell surface of both *A. baumannii* and *A. baylyi* ADP1 cells^23–25^. Only a relatively few STs host CDI system in *A. baumannii*, the same holds for T6SS cluster and *asaA* gene, whether there is a correlation between the presence/absence of T6SS and CDI system need further investigation.

In conclusion, we confirmed that AsaA is required for the secretion of Hcp and it most likely affects the assembly or stability of the T6SS by interacting with the periplasmic domain of the core T6SS protein TssM in the periplasmic space. We cannot exclude the possibility that AsaA associates with assisting the apparatus assembling or affecting the apparatus stability rather than as a T6SS structural component. Nonetheless, given the fact that AsaA is an *Acinetobacter* genus-specific protein, the results suggest that the T6SS structural components of *Acinetobacter* is distinctive from other Gram-negative pathogens.

## Methods

### Strains and culture conditions

All strains and plasmids are summarized in Table S1. The reporter strains were grown in M9 His-dropout medium, other strains were grown in LB medium. Antibiotics, when appropriate, were added to bacterial cultures medium at the following final concentrations in μg/mL: chloramphenicol (Cm) at 12.5, streptomycin (Sm) at 10, ampicillin (Amp) at 100, tetracycline (Tc) at 20, and kanamycin (Kan) at 50.

### Construction of mutant and complement strains

All primers used in this study are summarized in Tables S2. *asaA* and *tssM* gene knock out mutants of *A*. *baumannii* ATCC 17978 were constructed with the method described by Tracy and associates^26^, with modifications. Briefly, a kanamycin resistance cassette was amplified with a pair of 76 bp oligonucleotide primers with 56 nucleotides of homology to the flanking regions of the targeted gene and an additional 20 nucleotides of homology to kanamycin resistance cassette from plasmid pKD4^26^. Subsequently, the PCR products were electroporated into *A*. *baumannii* ATCC 17978 carrying pKD46 plasmid, then clones were screened on kanamycin containing LB agar plates^12^. Genomic DNA was isolated from kanamycin resistant clones, amplicons were prepared using primer set 2 and confirmed by sequencing. To remove the kanamycin resistance cassette, pCP20 expressing recombinase was transformed into the mutants. The *asaA* and *tssM* gene knock out mutants were confirmed by polymerase chain reaction (PCR) and sequencing.

For genetic complementation of mutant strains, the ORF (open reading frame) sequence of *asaA* (gene number: *A1S_1292*) and *tssM* (gene number: *A1S_1302*) was amplified from *A*. *baumannii* ATCC 17978. Subsequently, the PCR products were cloned into pTrc99A with confirmation by sequencing.

### Bacterial growth assays

Bacterial growth assays was performed as previously described by Salomon and associates^27^. Triplicates of *A*. *baumannii* strains grown overnight in LB were normalized to OD_600_ of 0.01, and the growth was monitored in LB of cultures incubated at 37 °C. Experiments were repeated at least twice with similar results. A representative experiment was shown.

### Bacterial competition assays

Bacterial competition assays was performed as previously described by Brent and associates^12^. The pk18*mob* plasmid was transformed into *E*. *coli* strain JM109, generating the strain named JM109/pK18*mob*. Cultures of *A*. *baumannii* and JM109/pK18*mob* were grown overnight, and the JM109/pK18*mob* was washed three times with PBS to remove kanamycin^12^. Cultures were diluted to OD_600_ of 1.0. Then 100 µl *E*. *coli* was mixed with 10 µl *A*. *baumannii*, and 10 µl of the mixture was spotted onto a LB agar plate. After 12 h incubation at 37 °C, spots were excised from the LB agar, the bacteria were diluted serially with 10-fold. Dilutions were plated onto kanamycin containing LB agar plates to select for *E*. *coli* and onto non-antibiotic resistant LB agar plates to determine the number of total bacteria^20^. Experiments were repeated at least twice with similar results. A representative experiment was shown.

### RNA isolation and semi-quantitative reverse transcription PCR (semi-quantitative RT-PCR)

All strains were grown to mid-logarithmic phase and total RNA was extracted using the RNAprep pure (Tiangen) and treated with RNase-free DNase according to the manufacturer’s protocol (Tiangen)^28^. Extracted total RNA was frozen in RNase-free water at −70 °C. RNA purity and concentration were determined using gel electrophoresis and spectrophotometer (NanoDrop, Thermo Scientific)^29^. Semi-quantitative RT-PCR was performed according to the One Step RNA PCR Kit instructions (Takara). Two sets of primers against *hcp* (*A1S_1296*) and 16S rRNA (*A1S_2837*) were designed using Vector NTI. For all primer sets, the following cycling parameters were used: 50 °C for 30 min, 94 °C for 2 min followed by 20 cycles of 94 °C for 30 sec, 55 °C for 30 sec, 72 °C for 40 sec. For standardization of results, the relative abundance of 16S rRNA was used as the internal standard^30^.

### Hcp secretion assays

pTrc99A-hcp recombinant vector expressing His-tagged Hcp was transformed into *A*. *baumannii* ATCC 17978. Cultures were grown in LB with ampicillin to exponential phase. Expression of Hcp was induced by 1 mM IPTG for 2 h. Then 10 ml cultures were centrifuged at 10 000 × *g* for 10 min to obtain whole cell pellets. The supernatant containing secreted Hcp protein were filtered through a 0.22 μm pore filter. A mixture of 8.5 ml of supernatant and 1.5 ml of 100% trichloroacetic acid (TCA) was placed on ice for 4 h and centrifuged at 10 000 × *g* for 20 minutes at 4 °C. Precipitated proteins were washed with 100% acetone and then re-suspended in 5 ml PBS. Subsequently, protein concentrations were measured using a BCA protein assay kit. Equal amounts of total protein (25 μg) were used for polyacrylamide gel electrophoresis and proteins were analyzed by Western blot^31^. Experiments were repeated at least twice with similar results. A representative experiment was shown.

### Cellular localization of AsaA

The cell fractionations were prepared as described by Zang *et al*.^32^ and Deng *et al*.^33^, with minor modifications. Briefly, overnight cultures of the cells were re-cultured with 1:10 and grown until an OD_600_ of 0.5 was reached. And then, the cells were collected by centrifugation at 4 000 × *g* for 10 min and the supernatant was filtered through a 0.22 μm pore filter and reserved as the extracellular proteins. The cell pellets were washed three times and re-suspended in 1 ml of periplasting buffer (30 000 U of lysozyme, 20% sucrose, 1 mM EDTA) and incubated on ice for 10 min. After centrifugation, the pellet was re-suspended in 10 mM magnesium chloride and incubated at 30 °C for 5 min, and then incubated at 0 °C for 10 min. The sample was freeze-thawed and pelleted by centrifugation, and then the supernatant was reserved as the periplasmic proteins. The cell was disrupted by sonication and further centrifuged at 11 000 × *g* for 30 min. The supernatant was then centrifuged at 130 000 × *g* for 1.5 h to isolate total membranes. The pellet was suspended in 0.25% (w/v) sodium lauryl sarcosine and centrifuged at 130 000 × *g* for 1.5 h. The supernatant was reserved as the inner membrane proteins. The pellet was re-suspended in TM buffer (8 mM MgSO_4_, 10 mM Tris) and reserved as the outer membrane proteins. All samples were analyzed by Western blot analysis by probing with antibodies to the 6× His tag (Hcp, AsaA, OmpA, DsbA and PglC) and DnaK^34^.

### Western blot

Proteins were resolved onto PVDF membranes. Membranes were blocked in 5% non-fat milk in 1× TBST buffer for 1 h at room temperature. Membranes were incubated overnight at 4 °C with anti-His_6_ or anti-DanK at a 1:1000 dilution. After washed with 1× TBST buffer for five times, the membrane was incubated with a HRP-conjugated secondary antibody for 50 min at room temperature. Membranes were then washed five times in 1× TBST buffer. Hybridizing bands were detected using the ECL kit.

### Bacterial two-hybrid assays

Bacterial two-hybrid assays *in vivo* were tested using the BacterioMatch^®^ II two-hybrid system (Stratagene). The truncated *asaA*, *tssM* and other genes were amplified by PCR with corresponding primers in Table S2. The amplified *asaA* gene excluding the N-terminal leader was fused to the vector pBT, yielding the plasmid pBA. The amplified *tssM* and other genes and their truncated derivatives were individually fused to the vector pTRG, respectively, yielding the plasmids pTM_1302(33–415)_, pTM_1302(436–1041)_, pTB, pTM_1303_ and pTL (Table S1).

In order to explore the interaction between AsaA and TssM, 120, 240, 360, 480 bp and other fragments containing partial *asaA* were fused to pBT, respectively. Pairs of plasmids were co-transformed into XL1-Blue MRF′, and the transformants were cultured on M9 His-dropout medium plate containing Sm (12.5 μg/ml) and 3-AT (5 mM) at 30 °C for 24 h.

### Overproduce and purification of recombinant protein

To overproduce the truncated peptides of AsaA and TssM, fragments coding sequence were fused to pET-30a. Recombinant plasmids were transformed into BL21(DE3) cell (Table S1). The recombinant strains were grown to an OD_600_ of 0.4, and then 1 mM IPTG was added to induce the expression of individual proteins. Cells were harvested and washed with PBS buffer, and broken by sonication. The recombinant proteins were purified by Ni-NTA His-bind^®^ (Novagen), as described in the manual.

### Pull-Down assays

To analyze the interaction between AsaA with TssM, pull-down assays were tested using the ProFound^TM^ pull-down biotinylated protein-protein interaction kit (Pierce). As described by li and associates^35^, the AsaA protein was biotinylated by sulfo-NHS-LC-biotin. Subsequently, the biotinylated AsaA was incubated with streptavidin sepharose^TM^ beads. The beads were washed four times and sample containing 60 µg of TssM protein was added and incubated. The beads were washed four times with washing buffer. Subsequently, the final protein was eluted by elution buffer. Finally, the final protein was separated by electrophoresis followed by coomassie blue staining.

## Supplementary information

Supplementary Information
