# Supplementary material for: The type VI secretion system protein AsaA in Acinetobacter baumannii is a periplasmic protein physically interacting with TssM and required for T6SS assembly
Source: Sci Rep. 2019 Jul 1;9:9438. doi: 10.1038/s41598-019-45875-9 (PMC6602968; doi:10.1038/s41598-019-45875-9)
Supplement: Supplementary file 1 — Supplementary Information [file 41598_2019_45875_MOESM1_ESM.pdf]

Supplementary information

Title: The type VI secretion system protein AsaA in *Acinetobacter baumannii* is a periplasmic protein physically interacting with TssM and required for T6SS assembly

Authors: Lei Li, Yi-Nuo Wang, Hong-Bing Jia, Ping Wang, Jun-Fang Dong, Juan Deng, Feng-Min Lu & Qing-Hua Zou

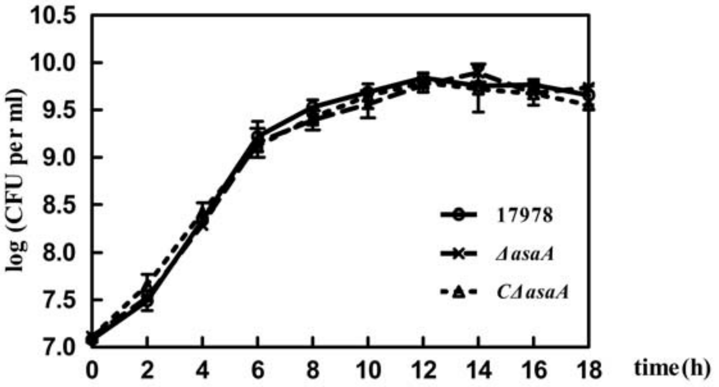

Supplementary Figure 1. A. baumannii strains growth curve in LB medium. The wild type 17978, AsaA mutant and complementation strains were used to inoculate LB medium at 10^7 CFU per ml. Samples were taken in triplicate at intervals of 2 h, diluted and plated on LB plates. Bacterial CFU were counted after incubation for 2 days. The experiments were repeated twice with similar results, and one representative result is presented.

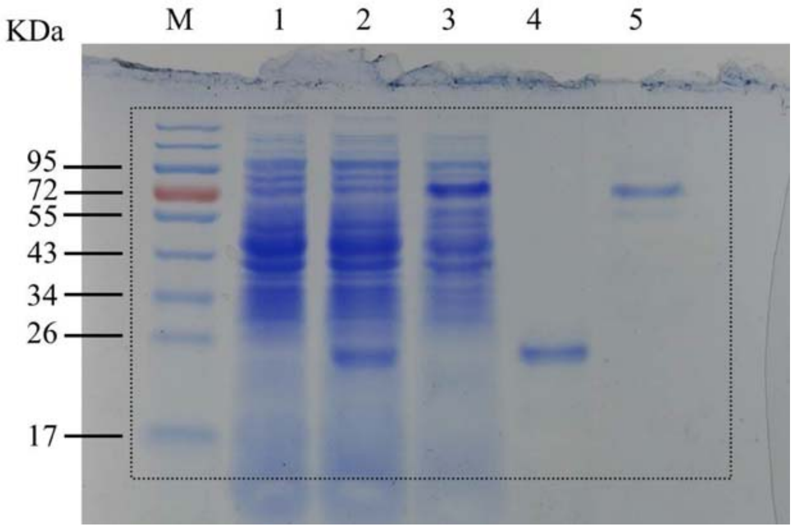

Supplementary Figure 2. The full-length gel of His_6-tagged fusion proteins were over expressed and purified. Lanes: 1, crude BL21/pET30a extract; 2, crude BL21/pET30a-AsaA extract induced with IPTG; 3, crude BL21/pET30a-$TssM_{436-1041}$ extract induced with IPTG; 4, affinity-purified His_6-AsaA protein; 5, affinity-purified His_6-$TssM_{436-1041}$ protein; M, molecular mass marker. Inside the box is the figure 4B.

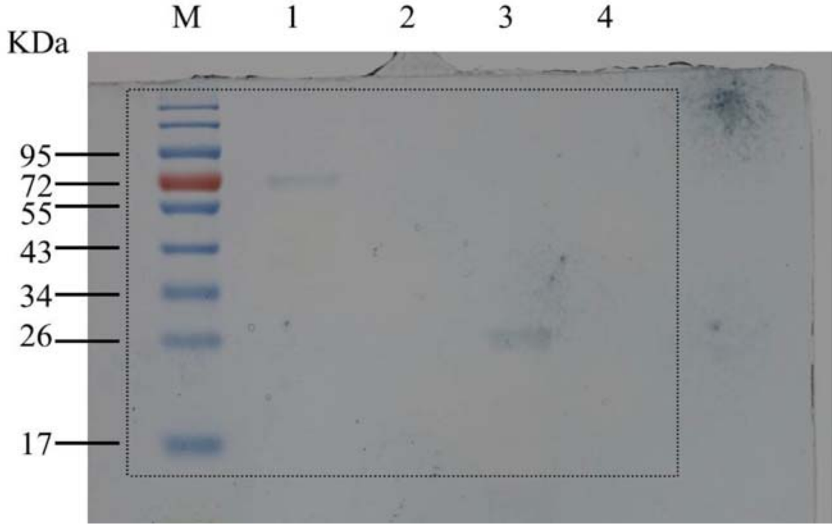

Supplementary Figure 3. The full-length gel of Pull-down assays. Lanes: 1, pull-down of His6-TssM by immobilized His6-AsaA; 2, His6-TssM mixed with streptavidin sepharose beads(negative control); 3, pull-down of His6-AsaA by immobilized His6-TssM; 4, His6-AsaA mixed with streptavidin sepharose beads(negative control) ; M, molecular mass marker. Inside the box is the figure 4C.

Table S1. Strains and plasmids used in this study

Strains or plasmids	Relevant characteristics	Reference or source
E. coli strains		
JM109	*RecA*1, *endA*1, *gyrA*96, *thi, supE*44, *relA*1 ⊿ (*lac-proAB*)/F′ [*traD36*, *lacI*^{q}, *lacZ* ΔM15]	[1]
JM109/pk18*mob*	JM109 harboring recombinant plasmid pk18*mob*, Kan^r	This work
BL21(DE3)	F$^-$ *ompT gal dcm lon hsdS*$_B$ ($r^-_B m^-_B$) λ(DE3)	Novagen,Germany
BL21/ pET30a	BL21(DE3) harboring plasmid pET30a, Kan^r	This work
BL21/pET30a-AsaA	BL21(DE3) harboring plasmid pET30a-AsaA, Kan^r	This work
BL21/pET30a-TssM$_{34-370}$	BL21(DE3) harboring plasmid pET30a-TssM$_{34-370}$, Kan^r	This work
XL1-Blue MRF′	Reporter strain, *Δ(mcrA)183 Δ(mcrCB-hsdSMR-mrr)173 endA1 hisB sup E44 thi1 recA1 gyrA96relA1 lac* [F′ lacI^q *HIS3 aadA* Kan^r]	Stratagene
X/pBA-pTB	XL1-Blue MRF′ harboring plasmid pBA and pTB, Kan^r Tc^r Cm^r	This work

X/pBA-pTL	XL1-Blue MRF′ harboring plasmid pBA and pTL, Kan^r Tc^r Cm^r	This work
X/pBA-pTM$_{1303}$	XL1-Blue MRF′ harboring plasmid pBA and pTM$_{1303}$, Kan^r Tc^r Cm^r	This work
X/pBA-pTM$_{33\text{-}415}$	XL1-Blue MRF′ harboring plasmid pBA and pTM$_{33\text{-}415}$, Kan^r Tc^r Cm^r	This work
X/pBA-pTM$_{436\text{-}1041}$	XL1-Blue MRF′ harboring plasmid pBA and pTM$_{436\text{-}1041}$, Kan^r Tc^r Cm^r	This work
X/pBA-pT	XL1-Blue MRF′ harboring plasmid pBA and pTRG, Kan^r Tc^r Cm^r	This work
X/pB-pTM$_{436\text{-}1041}$	XL1-Blue MRF′ harboring plasmid pBT and pTM$_{436\text{-}1041}$, Kan^r Tc^r Cm^r	This work
X/pBhpaM-pThrcJ	XL1-Blue MRF′ harboring plasmid pBhpaM and pThrcJ, Kan^r Tc^r Cm^r	[2]
X/pBA$_{25\text{-}70}$-pTM$_{436\text{-}1041}$	XL1-Blue MRF′ harboring plasmid pBA$_{25\text{-}70}$ and pTM$_{436\text{-}1041}$, Kan^r Tc^r Cm^r	This work
X/pBA$_{25\text{-}110}$-pTM$_{436\text{-}1041}$	XL1-Blue MRF′ harboring plasmid pBA$_{25\text{-}110}$ and pTM$_{436\text{-}1041}$, Kan^r Tc^r Cm^r	This work
X/pBA$_{25\text{-}150}$-pTM$_{436\text{-}1041}$	XL1-Blue MRF′ harboring plasmid pBA$_{25\text{-}150}$ and pTM$_{436\text{-}1041}$, Kan^r Tc^r Cm^r	This work
X/pBA$_{25\text{-}190}$-pTM$_{436\text{-}1041}$	XL1-Blue MRF′ harboring plasmid pBA$_{25\text{-}190}$ and pTM$_{436\text{-}1041}$, Kan^r Tc^r Cm^r	This work
X/pBA$_{25\text{-}230}$-pTM$_{436\text{-}1041}$	XL1-Blue MRF′ harboring plasmid pBA$_{25\text{-}230}$ and pTM$_{436\text{-}1041}$, Kan^r Tc^r Cm^r	This work
X/pBA$_{110\text{-}230}$-pTM$_{436\text{-}1041}$	XL1-Blue MRF′ harboring plasmid pBA$_{110\text{-}230}$ and pTM$_{436\text{-}1041}$, Kan^r Tc^r Cm^r	This work
X/pBA$_{150\text{-}230}$-pTM$_{436\text{-}1041}$	XL1-Blue MRF′ harboring plasmid pBA$_{150\text{-}230}$ and pTM$_{436\text{-}1041}$, Kan^r Tc^r Cm^r	This work
X/pBA$_{190\text{-}230}$-pTM$_{436\text{-}1041}$	XL1-Blue MRF′ harboring plasmid pBA$_{190\text{-}230}$ and pTM$_{436\text{-}1041}$, Kan^r Tc^r Cm^r	This work
X/pBA$_{70\text{-}150}$-pTM$_{436\text{-}1041}$	XL1-Blue MRF′ harboring plasmid pBA$_{70\text{-}150}$ and pTM$_{436\text{-}1041}$, Kan^r Tc^r Cm^r	This work
X/pBA$_{70\text{-}110}$-pTM$_{436\text{-}1041}$	XL1-Blue MRF′ harboring plasmid pBA$_{70\text{-}110}$ and pTM$_{436\text{-}1041}$, Kan^r Tc^r Cm^r	This work
X/pBA$_{110\text{-}150}$-pTM$_{436\text{-}1041}$	XL1-Blue MRF′ harboring plasmid pBA$_{110\text{-}150}$ and pTM$_{436\text{-}1041}$, Kan^r Tc^r Cm^r	This work
A. Baumannii strains		
17978	wild type strain	[3]
ΔasaA	As 17978, but *asaA* gene (*A1S_1292*) deleted.	This work
ΔtssM	As 17978, but *tssM* gene (*A1S_1302*) deleted.	This work
CΔasaA	ΔasaA harboring the recombinant plasmid pTrc99AasaA , Amp^r	This work
17978/pThcpH6	17978 harboring recombinant plasmid pTHcpH6, Amp^r	This work
ΔasaA /pThcpH6	*ΔasaA* harboring recombinant plasmid pTHcpH6, Amp^r	This work
ΔtssM /pThcpH6	*ΔtssM* harboring recombinant plasmid pTHcpH6, Amp^r	This work
ΔasaA / pTasaAH6	*ΔasaA* harboring the recombinant plasmid pTrc99AasaAH6 , Amp^r	This work
17978/pTpglCH6	17978 harboring recombinant plasmid pTpglCH6, Amp^r	This work
17978/pTompAH6	17978 harboring recombinant plasmid pTompA H6, Amp^r	This work
17978/pTdsbAH6	17978 harboring recombinant plasmid pTdsbAH6, Amp^r	This work
Plasmids		
pET30a	Expression vector, allow the production of fusion proteins containing amino terminal 6×His -tagged sequences. Kan^r	Novagen
pET30a-AsaA	pET30a containing a 618-bp fragment of partial *asaA* gene sequence encoding the 25th–230th amino acids. Kan^r	This work
pET30a- TssM$_{436\text{-}1041}$	pET-30a containing a 1818-bp fragment of partial *tssM* gene sequence encoding the 436th–1041th amino acids. Kan^r	This work
pTrc99A	P*trc*, pBR322ori, *rrnB* T1, *rrnB* T2, *lac* ^{Iq}, *bla*, template for P*trc* Promoter, Amp^r	[4]
pThcpH6	pTrc99A containing the encoding sequence of Hcp with 6×His tag in its C-terminus, Amp^r	This work

pTasaAH6	pTrc99A containing the encoding sequence of AsaA with 6×His tag in its C-terminus, Amp^r	This work
pTpglCH6	pTrc99A containing the encoding sequence of PglC with 6×His tag in its C-terminus, Amp^r	This work
pTompAH6	pTrc99A containing the encoding sequence of OmpA with 6×His tag in its C-terminus, Amp^r	This work
pTdsbAH6	pTrc99A containing the encoding sequence of DsbA with 6×His tag in its C-terminus, Amp^r	This work
pBT	Two-hybrid system bait plasmid containing the *cm* gene, p15A origin of replication and λcI ORF, Cm^r	Stratagene
pBA	pBT derivative carrying a 621-bp fragment encoding the 25th–230th amino acids of AsaA, Cm^r	This work
$pBA_{25\text{-}70}$	pBT derivative carrying a 138-bp fragment encoding the 25th–70th amino acids of AsaA, Cm^r	This work
$pBA_{25\text{-}110}$	pBT derivative carrying a 258-bp fragment encoding the 25th–110th amino acids of AsaA, Cm^r	This work
$pBA_{25\text{-}150}$	pBT derivative carrying a 378-bp fragment encoding the 25th–150th amino acids of AsaA, Cm^r	This work
$pBA_{25\text{-}190}$	pBT derivative carrying a 498-bp fragment encoding the 25th–190th amino acids of AsaA, Cm^r	This work
$pBA_{70\text{-}230}$	pBT derivative carrying a 483-bp fragment encoding the 70th–230th amino acids of AsaA, Cm^r	This work
$pBA_{110\text{-}230}$	pBT derivative carrying a 363-bp fragment encoding the 110th–230th amino acids of AsaA, Cm^r	This work
$pBA_{150\text{-}230}$	pBT derivative carrying a 243-bp fragment encoding the 150th–230th amino acids of AsaA, Cm^r	This work
$pBA_{190\text{-}230}$	pBT derivative carrying a 123-bp fragment encoding the 190th–230th amino acids of AsaA, Cm^r	This work
$pBA_{70\text{-}150}$	pBT derivative carrying a 243-bp fragment encoding the 70th–150th amino acids of AsaA, Cm^r	This work
$pBA_{70\text{-}110}$	pBT derivative carrying a 123-bp fragment encoding the 70th–110th amino acids of AsaA, Cm^r	This work
$pBA_{110\text{-}150}$	pBT derivative carrying a 123-bp fragment encoding the 110th–150th amino acids of AsaA, Cm^r	This work
pTRG	Two-hybrid system target plasmid containing the *tet* gene, ColE1 origin of replication, and RNA polymerase α subunit ORF, Tc^r	Stratagene
pTB	pTRG derivative carrying the 150-bp of *tssB* gene, Tc^r	This work
pTL	pTRG derivative carrying the 648-bp of *tssL* gene, Tc^r	This work
pTM_{1303}	pTRG derivative carrying the 567-bp of *A1S_1303* gene, Tc^r	This work
$pTM_{33\text{-}415}$	pTRG derivative carrying a 1149-bp fragment encoding the 33th–415th amino acids of *tssM* gene, Tc^r	This work
$pTM_{436\text{-}1041}$	pTRG derivative carrying a 1818-bp fragment encoding the 436th–1041th amino acids of *tssM* gene, Tc^r	This work

References

1. Yanisch-Perron, C., Vieira, J. & Messing, J. Improved M13 phage cloning vectors and host strains: nucleotide sequences of the M13mp18 and pUC19 vectors. *Gene* **33**, 103–119(1985).
2. Li, L., Li, R. F., Ming, Z. H., Lu, G. T. & Tang, J. L. Identification of a novel type III

secretion-associated outer membrane-bound protein from *Xanthomonas campestris* pv. *campestris*. *Sci Rep* **7**, 42724 (2017).
3. Smith, M. G. *et al.* New insights into *Acinetobacter baumannii* pathogenesis revealed by high-density pyrosequencing and transposon mutagenesis. *Genes Dev* **21**, 601-614 (2007).
4. Amann, E., Ochs, B. & Abel, K.J. Tightly regulated Tac promoter vectors useful for the expression of unfused and fused proteins in *Escherichia coli*. *Gene* **69**, 301–315(1988).

Table S2. Primers used in this study

Primer	Nucleotide sequence (5′→3′)	The amplified fragment or the utilization
asaA-F	TATTCTAAAAATATTAACTTTTCGGTAAGGCCTTGAATAAATAAGAAAACAGAGGAGTGTAGGCTGGAGCTGCTTC	DNA fragment of 1581-bp of the kanamycin resistance ORF sequence and coding sequence fusing with *asaA* upstream and downstream encoded sequence. Used for construction of *asaA* deletion mutant.
asaA-R	TTCGCTGAAGCTTCTTTTGTACGCTTTCTCTCTTAGCCATAAGATTCTATTCCGGACATATGAATATCCTCCTTAG	
tssM-F	GGCAGTACATCACGAATCCTAAAGCAATTATCGCCTTGTCATTGTTCGTGGCATTGGTGTAGGCTGGAGCTGCTTC	DNA fragment of 1581-bp of the kanamycin resistance ORF sequence and coding sequence fusing with *tssM* upstream and downstream encoded sequence. Used for construction of *tssM* deletion mutant.
tssM-R	TGCCATTCCATTTGTAGGTGCAACATAGCGTTGGAAGTTCATAACAAATTGTGGGTCATATGAATATCCTCCTTAG	
asaA-CF	AAGAATTCAAATATTAACTTTTCGGTAAGG	DNA fragment of 759-bp DNA fragment spans 48 upstream to the stop codon of the *asaA* ORF sequence, which fusing with 6×His tag encoding sequences. Used for construction of *ΔasaA* complement and Western blot analysis.
asaA-CR	AAGGATCCTTAGTGGTGGTGGTGGTGGTGTTTAATTAAAGGGTCAAAAGG	
hcp-qF	ATATATACGTTGAGTTTCG	381-bp internal fragment of *hcp*, used for qRT-PCR.
hcp-qR	AACCGAATGCTTCTGTAG	
16s-F	GTGCCAGCAGCCGCGGT	846-bp internal fragment of the 16S rDNA sequence, used for qRT-PCR.
16s-R	GACGGGCGGTGTGTACA	
hcp-HF	ATGGATCCGACCACATTTCCAGCTGG	DNA fragment of 627-bp *hcp* coding sequence fusing with 6×His tag encoding sequences. Used for Western blot analysis.
hcp-HR	AGTCTAGATTAGTGGTGGTGGTGGTGGTGCGCTGCGTAAGAAGC	
pglC-HF	CCGAATTCGTGAATGAAAATATGAA	DNA fragment of 633-bp *pglC* coding sequence fusing with 6×His tag encoding sequences. Used for Western blot analysis.
pglC -HR	AAGGATCCTTAGTGGTGGTGGTGGTGGTGCTTAACAGAAGAAT	
dsbA-HF	CCGAATTC ATGAAAAAAATTGGTTTTGG	DNA fragment of 618-bp *dsbA* coding sequence fusing with 6×His tag encoding sequences. Used for Western blot analysis.
dsbA-HR	AAGGATCCTTAGTGGTGGTGGTGGTGGTGTTTTGCCTTACGTT	
ompA-HF	CCGAATTCATGAAATTGAGTCGTATT	DNA fragment of 1071-bp *ompA* coding sequence fusing with 6×His tag encoding sequences. Used for Western blot analysis.
ompA -HR	AAGGATCCTTAGTGGTGGTGGTGGTGGTGTTGAGCTGCTGCAG	
tssM$_{(33-415)}$-F	CCGGATCCCAGCGTAAAAAGCATGCT	1149-bp DNA fragment spans nucleotides 97 to 1245 bp of the *tssM* coding sequence, encoding the 33th - 415th amino acids. Used for bacterial two-hybrid assay.
tssM$_{(33-415)}$-R	AAGGATCCACGCTGACGCTTTTTGCT	
tssM$_{(436-1041)}$-F	CCGGATCCTCATATCGTAACAATCAA	1818-bp DNA fragment spans nucleotides 1306 to 3123 bp of the *tssM* coding sequence, encoding the 436th - 1041th amino acids. Used for bacterial two-hybrid assay.
tssM$_{(436-1041)}$-R	AAGAATTCAACTAATCCCCAAATCTT	

tssB-F<br>*tssB*-R	CCGGATCCGTGTTCAGACTTACATAT<br>AAGAATTCCTAAATCGACATTAATGA	150-bp DNA fragment of the *tssB* ORF sequence. Used for bacterial two-hybrid assay.
tssL-F<br>*tssL*-R	CCGGATCCATGTCACAATCTACAGGT<br>AAGAATTCAGGTAGCTCACGATGGAT	648-bp DNA fragment spans nucleotides 1 to 648 bp of the *tssM* coding sequence, encoding the 1th - 216th amino acids. Used for bacterial two-hybrid assay.
Tss1303-F<br>*Tss1303*-R	CCGGATCCATGGCAACAGGTGAGTTA<br>AAGAATTCTCATGGCTTAACTCCCGC	567-bp DNA fragment of the *A1S_1303* ORF sequence. Used for bacterial two-hybrid assay.
asaA-OF<br>*asaA*-OR	AAGGATCCCAAGCAGCAGAACTAGAG<br>CGAAGCTTTTATTTAATTAAAGGGTC	621-bp DNA fragment spans nucleotides 73 to 693 bp of the *asaA* coding sequence, encoding the 25th - 230th amino acids. Used for pull-down assay.
tssM-OF<br>*tssM*-OR	AAGGATCCTCATATCGTAACAATCAA<br>CCGTCGACTTAAACTAATCCCCAAATCTT	1821-bp DNA fragment spans nucleotides 1306 to 3126 bp of the *tssM* coding sequence, encoding the 436th - 1041th amino acids. Used for pull-down assay.
asaA-1F<br>*asaA*-1R	CCGAATTCACAAGCAGCAGAACTAGAG<br>AAGGATCCTTATTTAATTAAAGGGTC	621-bp DNA fragment spans nucleotides 73 to 693 bp of the *asaA* coding sequence, encoding the 25th - 230th amino acids. Used for bacterial two-hybrid assay.
asaA-2F<br>*asaA*-2R	CCGAATTCACAAGCAGCAGAACTAGAG<br>AAGGATCCATTTTTTTGAGTTGTATT	138-bp DNA fragment spans nucleotides 73 to 210 bp of the *asaA* coding sequence, encoding the 25th - 70th amino acids. Used for bacterial two-hybrid assay.
asaA-3F<br>*asaA*-3R	CCGAATTCACAAGCAGCAGAACTAGAG<br>AAGGATCCTAAGACATCTGTGATTAA	258-bp DNA fragment spans nucleotides 73 to 330 bp of the *asaA* ORF sequence, encoding the 25th - 110th amino acids. Used for bacterial two-hybrid assay.
asaA-4F<br>*asaA*-4R	CCGAATTCACAAGCAGCAGAACTAGAG<br>AAGGATCCATGAATATTTTCAACTAA	378-bp DNA fragment spans nucleotides 73 to 450 bp of the *asaA* ORF sequence, encoding the 25th - 150th amino acids. Used for bacterial two-hybrid assay.
asaA-5F<br>*asaA*-5R	CCGAATTCACAAGCAGCAGAACTAGAG<br>AAGGATCCAGCACTCACTTTATTACG	498-bp DNA fragment spans nucleotides 73 to 570 bp of the *asaA* ORF sequence, encoding the 25th - 190th amino acids. Used for bacterial two-hybrid assay.
asaA-6F<br>*asaA*-6R	CCGAATTCATCTAGAACATCAAATAAA<br>AAGGATCCTTATTTAATTAAAGGGTC	123-bp DNA fragment spans nucleotides 571 to 693 bp of the *asaA* ORF sequence, encoding the 190th - 230th amino acids. Used for bacterial two-hybrid assay.
asaA-7F<br>*asaA*-7R	CCGAATTCAACTGCAATGCCGGCTCCT<br>AAGGATCCTTATTTAATTAAAGGGTC	243-bp DNA fragment spans nucleotides 451 to 693 bp of the *asaA* ORF sequence, encoding the 150th - 230th amino acids. Used for bacterial two-hybrid assay.
asaA-8F<br>*asaA*-8R	CCGAATTCATTTTTATCTGGTGTGAGC<br>AAGGATCCTTATTTAATTAAAGGGTC	363-bp DNA fragment spans nucleotides 331 to 693 bp of the *asaA* ORF sequence, encoding the 110th - 230th amino acids. Used for bacterial two-hybrid assay.
asaA-9F<br>*asaA*-9R	CCGAATTCAGATTTATTAGCTAAAGCT<br>AAGGATCCTTATTTAATTAAAGGGTC	483-bp DNA fragment spans nucleotides 211 to 693 bp of the *asaA* ORF sequence, encoding the 70th - 230th amino acids. Used for bacterial two-hybrid assay.
asaA-10F<br>*asaA*-10R	CCGAATTCAGATTTATTAGCTAAAGCT<br>AAGGATCCATGAATATTTTCAACTAA	240-bp DNA fragment spans nucleotides 211 to 450 bp of the *asaA* ORF sequence, encoding the 70th - 150th amino acids. Used for bacterial two-hybrid assay.
asaA-11F<br>*asaA*-11R	CCGAATTCAGATTTATTAGCTAAAGCT<br>AAGGATCCTAAGACATCTGTGATTAA	120-bp DNA fragment spans nucleotides 211 to 330 bp of the *asaA* ORF sequence, encoding the 70th - 110th amino acids. Used for bacterial two-hybrid assay.

asaA-12F	CC<u>GAATTC</u>ATTTTTATCTGGTGTGAGC	120-bp DNA fragment spans nucleotides 331 to 450 bp of the *asaA* ORF sequence, encoding the 110th -150th amino acids. Used for bacterial two-hybrid assay.
asaA-12R	AA<u>GGATCC</u>ATGAATATTTTCAACTAA	

The underlined sequences indicate the restriction sites for *Eco*RI, *Hin*dIII, *Sal*I , *Xba*I and *Bam*HI, respectively.

Table S3. AsaA homologues in the family *Moraxellaceae*

Species	Gene	Signal peptide	Length (aa)	Identity(%) /similarity(%)	Function predicted	Sequence Types (ST)	Reference
Acinetobacter baumannii AC29	*BL01_13280*	1th-24th aa	230	100/100	signal peptide protein	195	[1]
Acinetobacter baumannii BJAB0868	*BJAB0868_01408*	1th-24th aa	230	100/100	hypothetical protein	218	[2]
Acinetobacter baumannii 1656-2	*ABK1_1741*	1th-24th aa	230	100/100	putative exported protein	423	[3]
Acinetobacter baumannii AB307-0294	*ABBFA_002240*	1th-24th aa	230	99.6/99.6	hypothetical protein	231	[4]
Acinetobacter baumannii AB0057	*AB57_1478*	1th-24th aa	230	99.1/99.6	hypothetical protein	207	[4]
Acinetobacter baumannii BJAB0715	*BJAB0715_01479*	1th-24th aa	230	99.1/99.6	hypothetical protein	642	[5]
Acinetobacter baumannii AbH12O-A2	*LX00_06400*	1th-24th aa	230	99.1/99.1	signal peptide protein	924	[6]
Acinetobacter pittii	*BDGL_000638*	1th-26th aa	229	90.9/92.6	hypothetical protein	1527	[7]
Acinetobacter calcoaceticus	*BUM88_06735*	1th-26th aa	229	87.8/92.2	hypothetical protein	1043	accession CP020000.1
Acinetobacter nosocomialis	*RR32_11560*	1th-24th aa	229	80.9/87.8	signal peptide protein	1162	accession CP010368.1
Acinetobacter baylyi	*ACIAD2693*	1th-22th aa	218	43.5/57.8	hypothetical protein		[8]

Acinetobacter indicus	*CTZ23_07445*	1th-22 th aa	222	33.3/46.8	hypothetical protein	[9]

Searching the AsaA homologues were carried out by blast the genome sequences in NCBI database with the AsaA sequence. No, no significant similarity protein (similarity>20%) was found within the genome sequence. Signal peptide predictions was carried with the SignalP program. Identity(%)/similarity(%) analysis was performed using the Align X program in Vector NTI software.

Reference

1. Zhu, L. *et al.* Complete genome analysis of three *Acinetobacter baumannii* clinical isolates in China for insight into the diversification of drug resistance elements. *PLoS One* **8**, e66584(2013).
2. Lean, S. S., Yeo, C. C., Suhaili, Z. & Thong, K. L. Comparative genomics of two ST 195 carbapenem-resistant *Acinetobacter baumannii* with different susceptibility to polymyxin revealed underlying resistance mechanism. *Front Microbiol* **6**, 1445 (2015).
3. Vallenet, D. *et al.* Comparative analysis of *Acinetobacters*: three genomes for three lifestyles. *PLoS One* **3**, e1805 (2008).
4. Merino, M. *et al.* Complete genome sequence of the multiresistant *Acinetobacter baumannii* strain AbH12O-A2, isolated during a large outbreak in spain. *Genome Announc* **2**,e01182-14 (2014).
5. Rogel-Hernandez, M. A. *et al.* Genome sequence of *Acinetobacter lactucae* OTEC-02, isolated from hydrocarbon-contaminated soil. *Genome Announc* **5**, e00400-00417 (2017).
6. Zhan, Y. *et al.* Genome sequence of *Acinetobacter calcoaceticus* PHEA-2, isolated from industry wastewater. *J Bacteriol* **193**, 2672-2673 (2011).
7. Jung, J., Baek, J. H. & Park, W. Complete genome sequence of the diesel-degrading *Acinetobacter* sp. strain DR1. *J Bacteriol* **192**, 4794-4795 (2010).
8. Barbe, V. *et al.* Unique features revealed by the genome sequence of *Acinetobacter* sp. ADP1, a versatile and naturally transformation competent bacterium. *Nucleic Acids Res* **32**, 5766-5779 (2004).
9. Vettath, V. K. *et al.* Complete genome sequence of *Acinetobacter indicus* type strain SGAir0564 isolated from tropical air collected in singapore. *Genome Announc* **6**, e00230-18 (2018).
10. Daligault, H. E. *et al.* Complete genome assembly of a quality control reference isolate, *Moraxella catarrhalis* strain ATCC 25240. *Genome Announc* **2**, e00938-14 (2014).
11. Moghadam, M. S. *et al.* Isolation and genome sequencing of four arctic marine *Psychrobacter* strains exhibiting multicopper oxidase activity. *BMC Genomics* **17**, 117 (2016).
12. Che, S. *et al.* Complete genome sequence of antarctic bacterium *Psychrobacter* sp. strain G. *Genome Announc* **1**, e00725-13 (2013).
